# Barriers and facilitators in providing oral health care to nursing home residents, from the perspective of care aides—a systematic review protocol

**DOI:** 10.1186/s13643-016-0231-7

**Published:** 2016-04-07

**Authors:** Matthias Hoben, Huimin Hu, Tianyuan Xiong, Angelle Kent, Nadia Kobagi, Minn N. Yoon

**Affiliations:** Knowledge Utilization Studies Program (KUSP), Faculty of Nursing, University of Alberta, 5-006 Edmonton Clinic Health Academy (ECHA), 11405 87 Avenue, Edmonton, AB T6G 1C9 Canada; Faculty of Nursing, University of Alberta, Edmonton, Alberta Canada; West China College of Stomatology, Sichuan University, Sichuan, China; Department of Cardiology, West China Hospital, Sichuan University, Chengdu, China; School of Dentistry, Faculty of Medicine & Dentistry, University of Alberta, Edmonton, Alberta Canada

**Keywords:** Oral health care, Nursing homes, Barriers and facilitators, Care aides, Quality of care, Quality improvement

## Abstract

**Background:**

Unregulated care aides provide up to 80 % of direct resident care in nursing homes. They have little formal training, manage high workloads, frequently experience responsive behaviours from residents, and are at high risk for burnout. This affects quality of resident care, including quality of oral health care. Poor quality of oral health care in nursing homes has severe consequences for residents and the health care system. Improving quality of oral health care requires tailoring interventions to identified barriers and facilitators if these interventions are to be effective. Identifying barriers and facilitators from the care aide’s perspective is crucial.

**Methods:**

We will systematically search the databases MEDLINE, Embase, Evidence Based Reviews—Cochrane Central Register of Controlled Trials, CINAHL, and Web of Science. We will include qualitative and quantitative research studies and systematic reviews published in English that assess barriers and facilitators, as perceived by care aides, to providing oral health care to nursing home residents. Two reviewers will independently screen studies for eligibility. We will also search by hand the contents of key journals, publications of key authors, and reference lists of all the studies included. Two reviewers will independently assess the methodological quality of the studies included using four validated checklists appropriate for different research designs. Discrepancies at any stage of review will be resolved by consensus.

We will conduct a thematic analysis of barriers and facilitators using all studies included. If quantitative studies are sufficiently homogeneous, we will conduct random-effects meta-analyses of the associations of barriers and facilitators with each other, with care aide practices in resident oral health care, and with residents’ oral health. If quantitative study results cannot be pooled, we will present a narrative synthesis of the results. Finally, we will compare quantitative findings to qualitative studies to identify hypothesized associations or effects not yet tested quantitatively.

**Discussion:**

This review will advance the development of effective strategies for improving quality of oral health care and highlight gaps in research on barriers and facilitators to providing oral health care to nursing home residents, as perceived by care aides.

**Systematic review registration:**

PROSPERO CRD42015032454

**Electronic supplementary material:**

The online version of this article (doi:10.1186/s13643-016-0231-7) contains supplementary material, which is available to authorized users.

## Background

An estimated 70 to 80 % of paid direct care to residents in North American nursing homes is provided by care aides, including the important task of oral health care. However, care aides are a non-professional, largely unregulated work force with little or no formal training [1–5]. Around 90 % of care aides are female, most are over 40 years old, many are foreign-born (21 % in the USA and 60 % in Canada), and almost half speak English as a second language [1, 4, 5]. Care aides often work multiple jobs, and the majority earn less than half of the national median annual earnings (based on US data, where this workforce is best profiled) [5]. Although care aides frequently experience verbal and physical aggressive behaviour from residents with dementia [1, 6], they have insufficient training in dementia care and in managing responsive behaviours of those residents [5]. Relationships and communications between care aides and regulated nurses are often difficult and conflict-laden, negatively impacting job satisfaction and provision of individualized care [7–9]. Workload is high for care aides, with frequent interruptions, and they spend over 40 % of their work time on tasks that last no longer than 3 min [10]. A high workload combined with lack of time for tasks is associated with reduced job satisfaction [11] and burnout [12], which negatively affects staff health and ultimately the quality of care [13].

Providing oral health care to nursing home residents is complex and challenging for all care providers. Care aides often lack knowledge and training in providing proper oral health care to residents [14–17]. More and more residents now enter nursing homes retaining some natural teeth and with more complex prostheses and bridges than in the past, leading to increased or different care needs [18]. Residents with dementia need extra assistance with oral health care, and their responsive behaviours frequently impose additional challenges for care aides [19].

Oral health care practices are sub-standard in nursing homes, including insufficient brushing of teeth or cleaning of dentures for residents needing assistance, using fingers instead of a toothbrush to perform mouth care, not wearing clean gloves during mouth care, using improper tools and substances for mouth care, not looking into residents’ mouths, and not removing food leftovers [20–22]. A Canadian study notes that in their most recent shift, 59 % of the care aides surveyed felt rushed when doing mouth care and 19 % left mouth care undone [23]. Norwegian studies report unacceptable oral hygiene for up to 50 % of dentate residents and up to 30 % of residents with dentures [24, 25]. The Norwegian studies agree with an Australian [26] study that oral hygiene is significantly worse for residents who need assistance with oral health care than for independent residents and worse for residents with responsive behaviours during mouth care than for residents without [24–26].

The consequences are severe. Poor oral health increases health care costs, reduces residents’ quality of life through unnecessary pain and suffering, and elevates the risk of malnutrition, aspiration pneumonia, atherosclerosis, and premature death [27–30]. Bad breath, changed dental aesthetics, and altered speech can affect self-image and self-esteem, with serious psychological and social consequences [31, 32]. Caries is present in 41–76 % of dentate nursing home residents [33–40]. Among all residents, 5–17 % have dental pain [36, 40, 41], 32–49 % need periodontal treatment [36, 37, 41], 66–74 % have gingivitis [36, 39], and 3.4 % report gum pain or discomfort [37]. Improved oral health care for nursing home residents is thus urgent. At any given time, around 350,000 older adults live in Canadian nursing homes [42]; the figure for the USA is over 1.3 million [43] and 2.9 million for Europe [44]. The proportion of older adults (65 years or older) who live in nursing homes in Western countries ranges between 3 and 8 % [44, 45], and the demand for nursing home care is expected to increase substantially [44, 46, 47].

Tailoring improvement interventions to previously identified barriers and facilitators is crucial to achieving a desired change [48–50]. Given the central role that care aides play in providing oral health care to nursing home residents, identifying barriers and facilitators for improvement as perceived by this provider group is paramount in designing effective strategies for better quality of oral health care. Our aim is to identify, critically evaluate, and synthesize the available research evidence on the barriers and facilitators, as perceived by care aides, to providing good oral health care to nursing home residents. Our primary research question is the following: Which barriers and facilitators to providing oral health care to nursing home residents do care aides report? Should we be able to identify studies that, in addition to reporting barriers and facilitators, also assess the association of these factors with care aides’ oral health care practices and/or residents’ oral health, we will address the following secondary research questions: How are care aide reported barriers and facilitators to providing oral health care to nursing home residents associated with (a) care aides’ oral health care practices and (b) residents’ oral health?

## Methods/design

### Review design

We will conduct a systematic mixed-methods synthesis of research [51]. Our review methods and presentation of results will follow the *Cochrane Handbook of Systematic Reviews of Interventions* [52] and the Preferred Reporting Items for Systematic Reviews and Meta-Analyses (PRISMA) guidelines [53]. This protocol followed the PRISMA-P reporting guidelines for systematic review protocols [54] (Additional file 1).

### Search strategy

We will search the databases MEDLINE, Embase, Evidence Based Reviews—Cochrane Central Register of Controlled Trials, CINAHL, and Web of Science. We developed a search strategy combining oral health-related terms with terms related to care providers and residents in residential long-term care facilities (nursing homes) and pre-tested the strategy with an expert scientific librarian for each database (see Additional file 2 for details). We will retrieve all findings available in the respective database without limiting language and year of publication. We will also select three to five key journals and eight to ten key authors based on the number and relevance of their published papers for our research topic. We will search key journal contents and key author publications by hand. Further, we will screen reference lists of the studies included to ensure that we retrieve all the studies relevant to this review.

### Data management

We will manage all references in Zotero—an open source literature management software that facilitates cloud-based online collaboration of researchers. We will import all search results including abstracts into Zotero, use this software for title and abstract screening, upload all full texts retrieved from the Zotero database, and carry out the full-text screening using this software. All review team members will receive training in using Zotero prior to the screening, and we will conduct calibration exercises as well as regular team meetings to discuss issues in order to improve the application of the inclusion and exclusion criteria.

### Inclusion and exclusion criteria

We will include all types of published studies listed in the databases searched (Table 1): articles published in peer-reviewed journals and “grey” literature such as non-peer-reviewed articles, textbooks, reports, and theses. We will limit our search to *empirical studies* (qualitative, quantitative, and mixed methods) that assess barriers and facilitators perceived by care aides to providing oral health care to nursing home residents. In addition to studies only focusing on barriers and facilitators as reported by care aides, we will also include studies quantitatively assessing the association between barriers or facilitators and care aides’ oral health care practices or residents’ oral health outcomes, if the study explicitly notes that it identified barriers and facilitators as perceived by care aides as a first step. Examples of indicators for care aides’ oral health care practices are the following:

- Proportion of residents on a care unit or in a facility who receive assistance with cleaning their teeth at least once a day
- Proportion of care aides on a care unit or in a facility who adhere to defined criteria for oral health best practice, such as taking out a resident’s dentures at least once a day, cleaning and rinsing them, and putting them back into the resident’s mouth
- Proportion of criteria for oral health best practice to which care aides on a care unit or in a facility adhere

**Table 1:** Inclusion and exclusion criteria

	Inclusion criteria	Exclusion criteria
Study type	• Primary, empirical, quantitative studies (survey studies, randomized controlled trials, non-randomized trials with or without control group, cohort or case control studies, cross-sectional studies) • Qualitative studies (qualitative interviews, focus groups, ethnographic observations, qualitative case studies) • Mixed-methods studies • Systematic reviews and meta-analyses	• Non-empirical work (editorials, opinion texts, theoretical discussions) • Non-systematic (selective) reviews. We will, however, screen reference lists of those reviews for eligible studies.
Study focus	• Barriers and facilitators, as perceived by care aides, to providing oral health care to nursing home residents	• Barriers and facilitators to providing oral health care to nursing home residents as perceived by persons other than care aides, such as the following: - Other care provider groups (nurses, allied health providers, dental professionals) - Managers (care managers, directors of care, facility administrators) - Researchers - Policymakers • Studies not empirically assessing barriers and facilitators to providing oral health care from the perspective of care aides
Setting	• Residential facilities that provide care for frail older adults over a prolonged time period (nursing homes, personal care homes, special or complex care homes, residential long-term care facilities, residential facilities, skilled nursing facilities, etc.) • Residential facilities providing care for less dependent residents (assisted living, supportive living)	• Residential facilities providing care for relatively healthy and independent residents (i.e. independent living facilities, such as retirement homes, senior housing) • Day or night care facilities • Hospitals, home care, primary care, care housing
Participants	• Formal, paid, unregulated care providers (care aides) providing oral health care in nursing homes	• Unpaid caregivers, volunteers, family members • Regulated care providers (nurses, allied health providers, dental professionals)

Examples of indicators for residents’ oral health outcomes are tooth decay, tooth status, periodontal issues, and oral hygiene status. The studies need to report a quantitative outcome (e.g. correlations, regression parameters, relative risks) to assess the association between identified barriers/facilitators and either care aides’ oral health care practices or residents’ oral health status.

We will include intervention studies with or without control groups if they (a) explicitly assess barriers and facilitators from the perspective of care aides as a first step and (b) evaluate the effects of interventions to address those barriers and facilitators. Control interventions can be either usual care (no control intervention) or any kind of placebo intervention, such as dissemination of written recommendations on how to improve oral health care for nursing home residents.

We will include studies conducted in nursing homes, settings that are referred to by multiple terms across countries and jurisdictions [55] but that can be defined by the following [55–57]:

- Accommodating mainly older people with complex health and care needs who cannot remain at home or in a supportive living environment
- Providing 24-h support and assistance with activities of daily living and nursing care
- Delivering health services over an extended time, often until resident death

We will also include studies conducted in assisted living facilities, i.e. residential facilities providing care to residents who require supportive care, as recent research indicates that residents living in these facilities have functional and health-related limitations similar to nursing home residents [58].

We will include only studies of care aides, also called health care aides; personal care attendants; personal support workers; and continuing care assistants—Hewko et al. [5] identified 56 different titles. We define care aides as formal, paid, unregulated care providers “who provide supportive services and personal assistance to disabled, elderly and/or ill (acute or chronic) individuals requiring either short-term aide or long-term support” [5].

### Study screening

After duplicates are removed from retrieved studies, two review team members will independently screen the titles and abstracts of all the studies for inclusion. Each reviewer will assign each study to one of three categories: inclusion, exclusion, or full text needed for decision. At each stage of study identification, the reviewers will resolve discrepancies by consensus. Full texts will be retrieved for all the studies included based on screening of their titles and abstracts and for the studies with insufficient information in titles or abstracts to decide on inclusion. Two review team members will screen full texts independently for inclusion. A hand search of key author publications will be carried out using the same inclusion strategy. One review team member will carry out the key journal hand search, and a second team member will independently check the studies included. Two team members will independently screen the reference lists of all the studies included for any additional relevant studies.

### Quality appraisal

Two members of the review team will independently assess the methodological quality of the studies (risk of bias). They will discuss discrepancies until consensus is reached. Results will be discussed in detail by the whole research team for each study. To evaluate study quality, we will use four validated checklists as appropriate to each study’s design, all of which were used and described in detail in previous systematic reviews [11, 59–62].

- Systematic reviews and meta-analyses—Assessment of Multiple Systematic Reviews (AMSTAR) tool [63]. AMSTAR is a reliable and valid instrument [64–66] that assesses study quality in the categories of definition of an a priori design, study selection and data extraction, literature search, inclusion and exclusion criteria, list of studies included and excluded, characteristics and scientific quality of studies included, appropriateness of conclusions and methods used to combine findings, publication bias, and conflict of interest.
- Clinical studies with or without a control group and with or without randomized allocation of participants—Quality Assessment Tool for Quantitative Studies (QATQS) [67]. The QATQS is a reliable and valid instrument [67, 68] that assesses studies for selection bias, study design, confounders, blinding, data collection methods, withdrawals and dropouts, intervention integrity, and analyses.
- Cross-sectional studies—Estabrooks’ Quality Assessment and Validity Tool for Cross-Sectional Studies. This tool, developed based on Cochrane guidelines [69] and other evidence-based criteria [70, 71], assesses the methodological quality of studies through 12 items in the categories of sampling, measurement, and statistical analyses.
- Qualitative studies—Critical Appraisal Skills Program (CASP) Qualitative Research Checklist [72]. This checklist assesses whether (a) research aims are clearly stated; (b) qualitative methodology, research design, recruitment strategy, and data collection methods are appropriate; (c) relationships between researchers and participants are adequately considered; (d) ethical issues are sufficiently addressed; (e) data analyses are sufficiently rigorous; (f) findings are clearly stated; and (g) research is valuable overall.

We will rate the overall quality of each study included with a scoring method developed by de Vet et al. [73] and used in those previous systematic reviews. We will calculate the ratio of the obtained score to the maximum possible score, which varies with the checklist used and the number of checklist items applicable. Based on this quality score with a possible range of 0–1, we will rank studies as weak (≤0.50), low moderate (0.51–0.66), high moderate (0.67–0.79), or strong (≥0.80).

### Data extraction

One team member will extract study details and record them in an Excel spreadsheet: first author, year of publication, title, journal (or type of study, e.g. thesis, report, textbook), country of study, study purpose(s), study design, study sample (numbers and types of facilities, care aides, and residents included), types and characteristics of interventions/strategies studied (including control conditions, if applicable), types and characteristics of barriers/facilitators, care aides’ oral health care practices, residents’ oral health, other outcomes assessed (including assessment tools, if applicable), and main results. A second team member will double-check the data extraction for each study, with discrepancies resolved by consensus.

### Analyses

We will first conduct a thematic analysis of all the studies included [74]. This step is to identify and cluster different types of barriers and facilitators. Next, we will assess how those barriers and facilitators are related to each other and how they are associated with care aides’ practices and with residents’ oral health. We will first review the available quantitative evidence on those associations (i.e. effect sizes of correlations, regression parameters, relative risks), then compare those findings to the qualitative studies included to identify hypothesized associations or effects that have not yet been tested quantitatively. We will statistically pool results of quantitative studies, using random-effects meta-analysis if a sufficient number of quantitative studies report similar outcomes. If so, we will use the *I*^2^ statistic [75, 76] including 95 % confidence intervals [77] to assess statistical heterogeneity (variation beyond chance) and inconsistency of study results. As *I*^2^ is non-linearly related to the ratio of between- and within-study variances and its expected value depends on the number of included studies (especially for less than ten included studies), we will in addition report *H*^2^ (including 95 % confidence intervals), a more robust and unbiased measure for heterogeneity [78]. We expect a rather small number of eligible studies, and we are aware that in this case homogeneity as indicated by each of the included tests may be due to undetected heterogeneity [79]. In case of homogeneity indicated by our homogeneity tests, we will therefore conduct sensitivity analyses, including moderate and large homogeneity assumptions in our models, and compare results to the model based on homogeneity assumption [79]. We will use random-effects models to assess study results, as those models are performing better than fixed effects models in case of heterogeneity and small numbers of included studies [80, 81]. We will also check if study protocols are available for the included studies (especially randomized controlled trials and systematic reviews) and if they were published before recruitment of participants started or before data extraction of included papers was completed, respectively. We will then compare the protocols to the published studies to assess if reporting bias is present. If we are able to include at least ten comparable studies, we will use funnel plots to assess publication bias. In any case, we will consider the year of publication of the included studies (i.e. number of studies included for each year of publication) to determine publication bias, as Kicinski et al. indicate that publication bias is more likely in older compared to more recent studies [82]. If the studies included are too heterogeneous to pool results statistically (e.g. different designs, settings, outcomes), we will construct a narrative synthesis of their outcomes. We will summarize the study designs used, the interventions and control interventions (if applicable) assessed, the resident and care aide outcomes studied, and the effect sizes found. Our pre-tests of the search strategies and our preliminary findings from the title and abstract screenings indicate that we will most likely not be able to synthesize study findings statistically because the number of eligible studies is too small and heterogeneity of study interventions and outcomes is too great.

## Discussion

This review will identify, synthesize, and critically evaluate published research studies that assess barriers and facilitators, as perceived by care aides, to providing better oral health care to nursing home residents. To achieve desired changes in oral health care practices, improvement interventions must be tailored to identified barriers and facilitators. Care aides are the main providers of oral health care to nursing home residents; thus, identifying barriers and facilitators from their perspective is crucial to improving quality of oral health care in nursing homes. This review will highlight gaps in the available research on barriers and facilitators to providing better oral health care, as seen by care aides. It will thereby advance the development of effective strategies to improve quality of oral health care for residents of nursing homes.

## Additional files

Additional file 1: PRISMA-P checklist. The checklist is composed of recommended items to address in a systematic review protocol. (PDF 218 kb) Additional file 2: Search strategy. The file gives a list of search terms/keywords to be used for this protocol. (PDF 133 kb)
