# Supplementary material for: Barriers and facilitators in providing oral health care to nursing home residents, from the perspective of care aides—a systematic review protocol
Source: Syst Rev. 2016 Apr 7;5:53. doi: 10.1186/s13643-016-0231-7 (PMC4823843; doi:10.1186/s13643-016-0231-7)
Supplement: Additional file 2: — Search strategy. The file gives a list of search terms/keywords to be used for this protocol. (PDF 133 kb) [file 13643_2016_231_MOESM2_ESM.pdf]

Supplementary file 1: Search strategy

MEDLINE 1946 to Present, MEDLINE(R) In-Process & Other Non-Indexed Citations, EBM Reviews - Cochrane Central Register of Controlled Trials (platform OVID)

1.	exp Dentistry/
2.	exp Tooth Diseases/
3.	Oral Health/
4.	Oral Hygiene/
5.	((oral or dental or mouth) adj (health or care or hygiene)).mp.
6.	or/1-5
7.	Nurses' Aides/
8.	(Care aide* or Care Attendant* or Care Guide* or paid caregiver* or Certified Nursing Assistant* or CNA* or Client Care Attendant* or Direct care worker* or Client Care Attendant* or Geriatric Health Aide* or Health Care Assistant* or Health Care Assistant* or Institutional Aide* or Medical Assistant* or Nurses aide* or Nursing assistant* or Nursing attendant* or Nursing home aide* or Personal Care Attendant*).tw.
9.	(Personal Care Nurse* or Personal Care Assistant* or Personal Care Attendant* or Personal support worker* or Residential Aide*).tw.
10.	(caregiver* and (nursing home* or residence* or residential or long term care or facility or facilities or institution*)).mp.
11.	or/7-10
12.	6 and 11
13.	Geriatric Nursing/ or nursing homes/ or Intermediate Care Facilities/ or skilled nursing facilities/ or homes for the aged/ or "Institutionalization"/
14.	(nursing adj (home* or center* or centre* or facilit*)).tw.
15.	((extended or long term or intermediate or skilled or assisted or supportive) adj care).tw.
16.	((extended or long term or intermediate or skilled or assisted or supportive) adj facilit*).tw. or ((assisted) adj living).tw
17.	((elderly or senior* or geriatric or veteran*) adj3 (institution* or home* or facilit* or unit* or center* or centre*)).tw.
18.	(rest adj2 home*).tw.
19.	convalescen* home*.tw.
20.	assisted care facilit*.tw.
21.	continuing care.tw.
22.	residential care.tw.
23.	or/13-22
24.	6 and 23

25.	exp Child/
26.	(child* or boy* or girl* or p?ediatric* or teen* or youth* or adolescen*).mp.
27.	25 or 26
28.	exp aged/
29.	(senior* or elder* or geriatric* or gerontolog*).mp.
30.	28 or 29
31.	27 and 30
32.	27 not 31
33.	24 not 32
34.	33 or 12
35.	remove duplicates from 34

CINAHL (platform EBSCOhost)

S1	(MH "Dentistry+") OR (MH "Tooth Diseases+") OR (MH "Oral Health") OR (MH "Oral Hygiene+") OR (MH "Dental Hygiene")
S2	(oral W0 (health or care or hygiene)) OR (dental W0 (health or care or hygiene)) OR (mouth W0 (health or care or hygiene))
S3	S1 OR S2
S4	(MH "Nursing Assistants") OR (MH "Nursing Home Personnel")
S5	("Care aide*" or "Care Attendant*" or "Care Guide*" or "paid caregiver*" or "Certified Nursing Assistant*") OR (CNA* or "Client Care Attendant*" or "Direct care worker*" or "Client Care Attendant*" or "Geriatric Health Aide*") OR ("Health Care Assistant*" or "Health Care Assistant*" or "Institutional Aide*" or "Medical Assistant*") OR ("Nurs* aide*" or "Nurs* assistant*" or "Nurs* attendant*" or "Nurs* home aide*" or "Personal Care Attendant*")
S6	"Personal Care Nurse*" or "Personal Care Assistant*" or "Personal Care Attendant*" or "Personal support worker*" or "Residential Aide*"
S7	(MH "Gerontologic Nursing+") OR (MH "Nursing Homes+")
S8	(MH "Nursing Home Patients") OR (MH "Institutionalization+")
S9	nursing W0 (home* or center* or centre* or facilit*)
S10	"extended care" or "long term care" or "intermediate care" or "skilled care" or "assisted care" or "assisted living" or "supportive care"
S11	(extended or "long term" or intermediate or skilled or assisted or supportive) W2 facilit*
S12	((elderly or senior* or geriatric or veteran*) N3 institution*) OR ((elderly or senior* or geriatric or veteran*) N3 home*) OR ((elderly or senior* or geriatric or veteran*) N3 facilit*) OR ((elderly or senior* or geriatric or veteran*) N3 unit*) OR ((elderly or senior* or geriatric or veteran*) N3 center*) OR ((elderly or senior* or geriatric or veteran*) N3 centre*)
S13	"rest home*" OR "convalescen* home*" OR "assisted care facilit*" OR :continuing care" OR "residential care"
S14	S4 OR S5 OR S6 OR S7 OR S8 OR S9 OR S10 OR S11 OR S12 OR S13
S15	S3 AND S14
S16	S3 AND S14<br>Limiters - Research Article
S17	senior* or older* or gerontolog* or geriatric* or elder*
S18	S16 AND S17
S19	S3 AND S14
S20	S18 OR S19

Web of Science Core Collection

#1	TS=("oral health" OR "oral care" OR "oral hygiene" OR dentistry OR "dental health" OR "dental care" OR "dental hygiene")
#2	TS=("Care aide*" or "Care Attendant*" or "Care Guide*" or "paid caregiver*" or "Certified Nursing Assistant*" or CNA* or "Client Care Attendant*" or "Direct care worker*" or "Geriatric Health Aide*" or "Health Care Assistant*" or "Health Care Assistant*" or "Institutional Aide*" or "Medical Assistant*" or "Nurs* aide*" or "Nurs* assistant*" or "Nurs* attendant*" or "Nurs* home aide*" or "Personal Care Attendant*" or "personal support worker*")
#3	TS=("nursing home*" or "residential care" or "continuing care" or facilit* or residence or residential or "long term care" or institution* or "assisted care" or "assisted living" "supportive care")
#4	#2 OR #3
#5	#1 AND #4
